# Genetic causality of lipidomic and immune cell profiles in ischemic stroke

**DOI:** 10.3389/fneur.2024.1437153

**Published:** 2024-09-30

**Authors:** Haohao Chen, Zequn Zheng, Xiaorui Cai, Shunxian Li, Manli Chen, Jiaming Wu, Wenzhen He, Fenfei Gao

**Affiliations:** ^1^Department of Pharmacy, The First Affiliated Hospital of Shantou University Medical College, Shantou, China; ^2^Department of Pharmacy, Shantou University Medical College, Shantou, China; ^3^Department of Cardiology, First Affiliated Hospital of Shantou University Medical College, Shantou, Guangdong, China; ^4^Clinical Research Center, First Affiliated Hospital of Shantou University Medical College, Shantou, Guangdong, China; ^5^The Affiliated Cancer Hospital of Shantou University Medical College, Shantou, China; ^6^Department of Neurology, The First Affiliated Hospital of Shantou University Medical College, Shantou, China

**Keywords:** ischemic stroke, large artery stroke, small vessel stroke, cardioembolic stroke, lipidomic, immune cell phenotypes, Mendelian randomization, genetic causality

## Abstract

**Background:**

Ischemic stroke (IS) is a global health issue linked to lipid metabolism and immune cell responses. This study uses Mendelian randomization (MR) to identify genetic risk factors for IS subtypes using comprehensive genetic data from lipidomic and immune cell profiles.

**Methods:**

We assessed genetic susceptibility to IS across 179 lipids and 731 immune cell phenotypes using instrumental variables (IVs) from recent genome-wide association studies. A two-sample MR approach evaluated correlations, and a two-step MR mediation analysis explored the role of immune cell phenotypes in the lipid-IS pathway. Sensitivity analyses, including MR-Egger and Cochran *Q* tests, ensured robust results.

**Results:**

Genetic IVs for 162 lipids and 614 immune cell phenotypes were identified. Significant genetic causality was found between 35 lipids and large artery stroke (LAS), with 12 as risk factors (sterol esters, phosphatidylcholines, phosphatidylethanolamines) and 23 as protective factors (phosphatidylcholines, phosphatidylethanolamines, phosphatidylinositols). For small vessel stroke (SVS), 8 as risk factors (sterol esters, phosphatidylcholines), and 2 as protective factors (phosphatidylinositol, sphingomyelin). For cardioembolic stroke (CS), 2 as risk factors, and 4 as protective factors. Mediation analysis revealed that CCR2 on granulocytes, CD11c on CD62L^+^ myeloid dendritic cells, and FSC-A on granulocytes mediated the lipid-immune cell-LAS pathway, while CD4 on activated CD4 regulatory T cells and CD4 on activated & secreting CD4 regulatory T cells mediated the lipid-immune cell-SVS pathway.

**Conclusion:**

This study identifies genetic links between specific lipids and IS subtypes, highlights immune cells’ role in IS risk and mediation, suggests new therapeutic targets, and uncovers IS genetic drivers.

## 1 Introduction

Ischemic stroke (IS) is a leading cause of morbidity and mortality worldwide, presenting a significant public health challenge. The pathogenesis of IS is complex and multifactorial, involving genetic, environmental, and metabolic factors (1–3). Among these, lipid metabolism and immune cell responses are crucial contributors to the onset and progression of IS (4–6).

Dyslipidemia, characterized by abnormal lipid levels in the blood, is a well-recognized modifiable risk factor for IS. Traditional lipid markers such as total cholesterol, triglycerides, low-density lipoprotein cholesterol (LDL-C), and high-density lipoprotein cholesterol (HDL-C) are commonly used to assess stroke risk (7, 8). However, advances in mass spectrometry have facilitated the development of lipidomics, allowing for the simultaneous detection of multiple lipids. This technological progress has significantly enhanced our understanding of the role of lipid metabolism in disease processes, including IS (9–11).

Additionally, immune cells are important for the inflammatory response associated with IS. Immune cell phenotypes, including granulocytes, dendritic, and T cells, participate in the inflammatory cascade that exacerbates brain damage following ischemic events (4, 12, 13). The interplay between lipid metabolism and immune cell function is a burgeoning area of research with important implications for identifying novel therapeutic targets and improving stroke prognosis.

We utilized Mendelian randomization (MR) techniques to investigate the genetic basis of lipid metabolism and immune cell responses in the context of IS. By using genetic variants as instrumental variables (IVs), MR provides a robust method to infer causality and mitigate confounding inherent in observational studies (14, 15). We performed a two-sample MR analysis of 179 lipidomic traits and three IS subtypes—large artery stroke (LAS), small vessel stroke (SVS), and cardioembolic stroke (CS)—using genetic data from genome-wide association studies (GWAS) (16, 17). Additionally, we conducted a two-step MR (TSMR) mediation analysis to examine whether immune cell phenotypes mediate the causal relationship between lipid profiles and IS risk (18, 19).

This study aims to identify specific lipid species and immune cell phenotypes that contribute to IS risk, elucidate potential causal pathways, and highlight novel therapeutic targets for the prevention and management of IS. By integrating comprehensive genetic data with advanced MR methodologies, we seek to deepen our understanding of the complex interactions between lipid metabolism, immune responses and IS.

## 2 Materials and methods

### 2.1 Study overview

**Figure 1:**
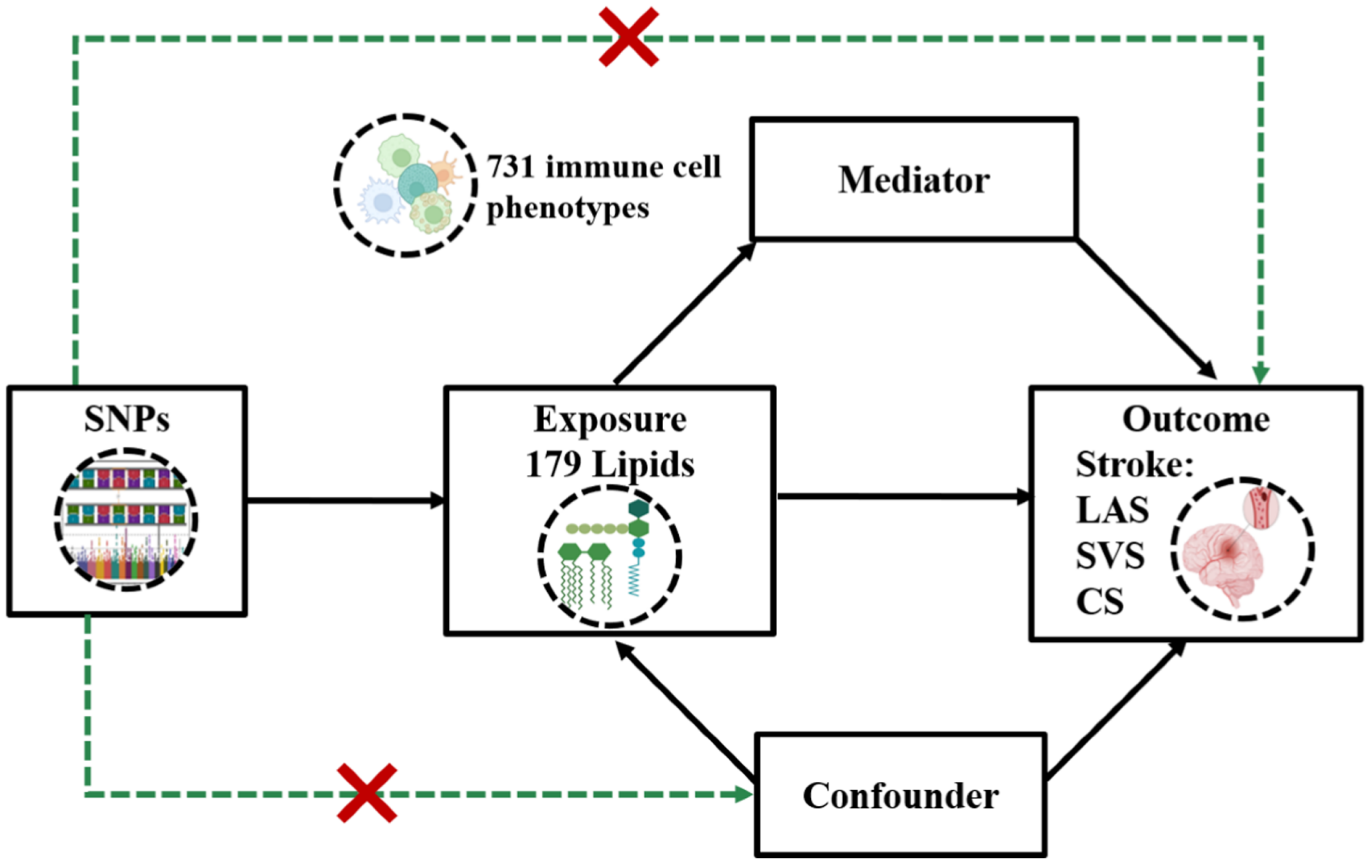
Assumptions and design of a two-step Mendelian randomization (TSMR) analyses. Firstly, a two-sample MR was performed to investigate the causal relationships between 179 lipid phenotypes and three distinct ischemic stroke subtypes. Secondly, 731 immune cell phenotypes were selected for subsequent mediation analyses. Finally, TSMR analysis was conducted to detect potential mediating immune cell phenotypes. TSMR, two-step Mendelian randomization; SNPs, single-nucleotide polymorphisms; LAS, large artery stroke; SVS, small vessel stroke; SC, cardioembolic stroke.

We performed a two-sample MR analysis utilizing genetic variants derived from the latest available GWAS of 179 plasma lipidomic and three subtypes of IS: LAS, SVS, and CS. We used inverse-variance weighted (IVW) and weighted median (WM) methods for the MR analyses, complemented by various sensitivity tests to ensure result robustness. Given the close relationship between immune cells and IS, we applied TSMR to determine if the identified effects were mediated through immune cell regulation. The first two-sample MR analysis examined 731 immune cell phenotypes as exposures and the three IS subtypes as outcomes. This was followed by a final two-sample MR analysis, where plasma lipids showing significant causality were used as exposures, and immune cell phenotypes with significant MR results in the GWAS were used as outcomes. The effectiveness of this MR approach depends on three key assumptions (1): genetic variants must strongly correlate with the exposure (2), variants influence the outcome only through the exposure, and (3) variants are free from confounding variables. The methodological workflow is depicted in Figure 1.

### 2.2 GWAS summary statistics

We accessed stroke-related data from the GIGASTROKE Consortium,^1^ which includes three IS subtypes: LAS with 9,219 cases and 1,496,931 controls, SVS with 13,620 cases and 1,496,931 controls, and CS with 12,828 cases and 1,496,931 controls (17). Additionally, we utilized GWAS data for 731 immune cell phenotypes, cataloged from GCST90001391 to GCST90002121 (18), and for 179 lipid traits, spanning from GCST90277238 to GCST90277416 (see text footnote 1) (16). Each dataset adheres to the ethical standards of the original studies, ensuring that no additional ethical approval was required for this secondary analysis. These datasets are detailed in Supplementary Table S1.

### 2.3 Selection of instrumental variables

To ascertain causal connections between lipidomic and immune cell profiles (exposure) and IS subtype outcomes, we employed genetic proxies, specifically SNPs, associated with these phenotypes. We selected SNPs associated with lipidomic and immune cell phenotypes using thresholds (*p* < 5 × 10^−8^) and applied clumping criteria with an LD *r*^2^ > 0.001 within a 10,000-kilobase window, based on the 1,000 Genomes European panel. To assess instrument strength and avoid weak instrument bias, we calculated the *F* statistic for each SNP, ensuring it was above 10, following the method outlined by Pierce and Burgess (20). Only SNPs exclusively related to the lipidomic and immune cell traits were included, ensuring no overlap with genes influencing ischemic stroke risk to adhere to the exclusion restriction criterion.

### 2.4 Statistical analysis

We initiated our investigation by conducting a two-sample MR analysis to investigate the causal relationship between lipidomic phenotypes and IS. The IVW and WM methods were employed for primary effect estimation. Analyses were conducted using a *p*-value threshold <0.05 to ascertain statistical significance, avoiding Bonferroni adjustments to preserve exploratory study objectives.

We utilized summary statistics of immune cell phenotypes, covering 731 immune cell levels in the blood, to explore potential immune cell levels as mediators between lipidomic profiles and ischemic stroke. We employed TSMR approach to delineate effects of lipidomic phenotypes and immune cell levels on IS subtypes. In addition to the estimation of the potential impact of lipidomic phenotypes on ischemic stroke derived from MR analyses (*β*_0_), two additional estimates were calculated (1): the causal effect of 731 immune cell levels on ischemic stroke (*β*_1_), and (2) the causal effect of exposure (lipid species significantly associated with IS subtypes) on the mediator (immune cell level species significantly associated with IS subtypes) (*β*_2_). Indirect effect, representing the causal effect of lipidomic profiles on IS subtypes via mediators, was estimated using the coefficient product method (*β*_1_ × *β*_2_). Mediation ratio was calculated as the “indirect effect/total effect” ([*β*_1_ × *β*_2_]/*β*_0_) (19).

### 2.5 Sensitivity analyses

For sensitivity analyses, we employed three MR methods: IVW, WM, and MR-Egger, each based on different assumptions about pleiotropy to generate effect estimates. Evidence of horizontal pleiotropy was suggested if the MR-Egger intercept significantly differed from zero (*p*-value <0.05). Heterogeneity was evaluated using the Cochran *Q* test, where a *p*-value greater than 0.05 indicated an absence of heterogeneity (21, 22). To corroborate the conclusions on causality, we ensured that: (a) the MR-Egger intercept did not show significant directional pleiotropy, and (b) Cochran’s *Q* test indicated no significant heterogeneity.

MR analyses were conducted in R (version 4.3.3; R Foundation for Statistical Computing, Vienna, Austria) with the “TwoSampleMR” packages.

## 3 Results

### 3.1 Causal associations between lipidomic profiles and IS subtypes

Our investigation probed the causal relationships between lipidomic profiles and IS subtypes, specifically focusing on three subtypes: LAS, SVS, and CS. We commenced by identifying IVs for 179 lipid species, ensuring each met the criteria for strong correlation and independence. IVs were successfully established for 162 lipid species, with *F*-statistic values ranging from 29.79 to 1946.15, effectively negating concerns of weak instrumental bias (Supplementary Table S2).

**Figure 2:**
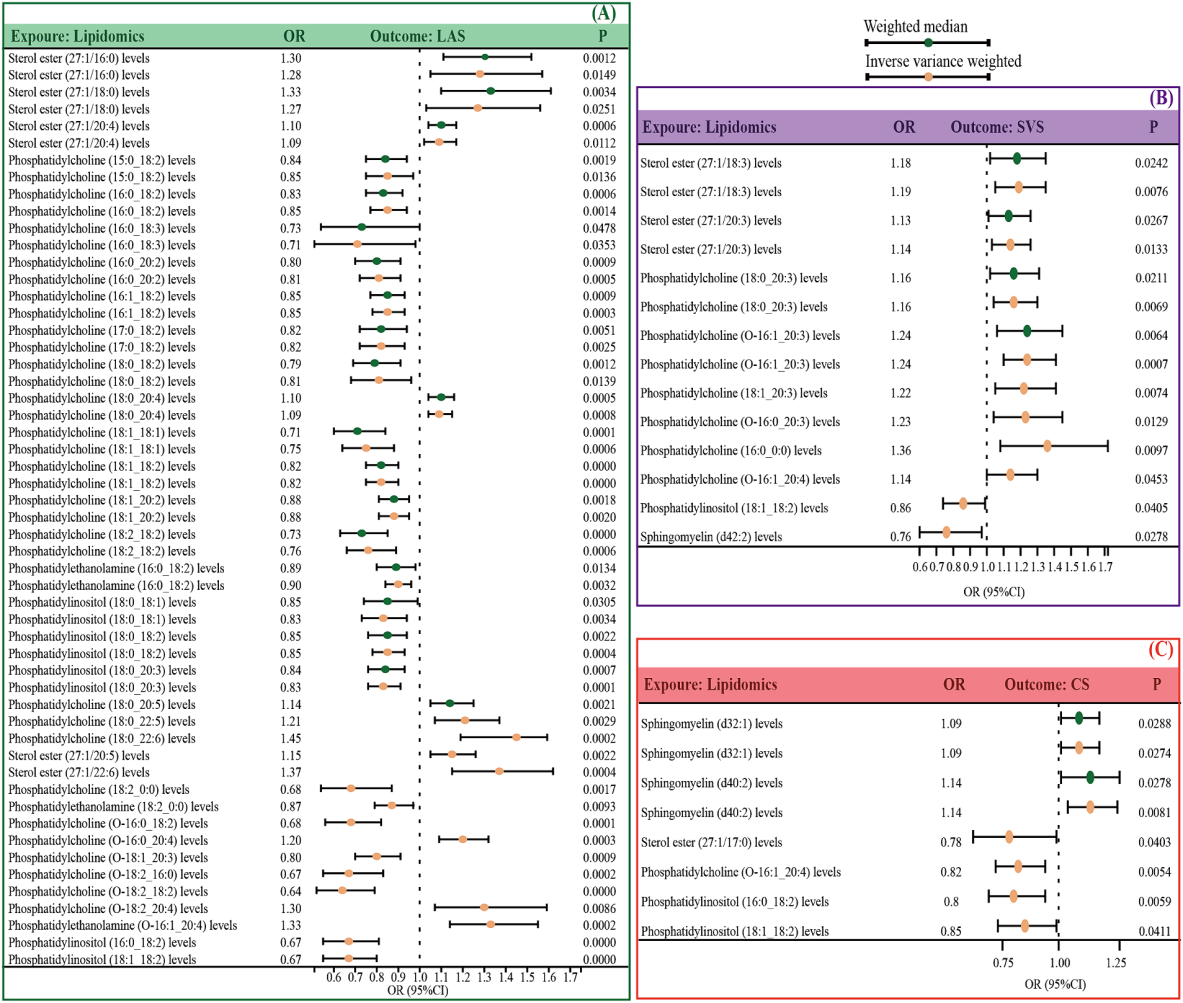
Significant MR estimates for specific lipids and IS subtypes (LAS, SVS, and CS) were assessed by IVW and WM. **(A)** The significant causal effect of lipids on LAS. **(B)** The significant causal effect of lipids on SVS. **(C)** The significant causal effect of lipids on CS. The dots colored in red and green indicate IVW and WM respectively. IS, ischemic stroke, IVW, inverse variance-weighted; MW, weighted median; MR, Mendelian randomization; OR, odds ratio; 95% CI, 95% confidence interval.

If the number of SNPs is greater than or equal to 3, we evaluated the data using both the IVW and WM methods; otherwise, only IVW was used. Our findings indicated that 35 lipid species are genetically causally associated with LAS; of these, 12 were identified as risk factors including 5 sterol esters, 6 phosphatidylcholines, and 1 phosphatidylethanolamine, and 23 as protective factors, comprising 16 phosphatidylcholines, 2 phosphatidylethanolamines, and 5 phosphatidylinositols (Figure 2A and Supplementary Table S3). Furthermore, 10 lipid species were associated with SVS, with 8 identified as risk factors (2 sterol esters and 6 phosphatidylcholines) and 2 as protective (1 phosphatidylinositol and 1 sphingomyelin) (Figure 2B and Supplementary Table S13). Additionally, 6 lipids demonstrated a causal association with CS, among which 2 sphingomyelins were risk factors, and 4 were protective including 1 sterol ester, 1 phosphatidylcholine, and 2 phosphatidylinositols (Figure 2C and Supplementary Table S22).

To assess and mitigate potential biases from directional horizontal pleiotropy in the MR results, Egger’s intercept test was conducted for phenotypes supported by more than three IVs. The *p*-values of the MR-Egger intercept estimates consistently exceeded 0.05, indicating no significant pleiotropy bias. Moreover, the Cochran Q test, indicating a *p*-value greater than 0.05 across all analyses, affirmed the absence of heterogeneity, thus substantiating the robustness of our causal inferences (Table 1; Supplementary Tables S4, S5, S14, S15, S23, S24).

**Table 1:** Pleiotropy and heterogeneity assessment for significant results (*p* < 0.05).

Trait.exposure	Trait.outcome	Directional pleiotropy		Cochran’s *Q* test	
		Egger_intercept	*p*-value	*Q*	*p*-value
Sterol ester (27:1/16:0) levels	LAS	−0.016	0.892	3.578	0.167
Sterol ester (27:1/18:0) levels		0.007	0.889	14.309	0.056
Sterol ester (27:1/20:4) levels		−0.039	0.118	6.105	0.191
Phosphatidylcholine (15:0_18:2) levels		0.014	0.634	9.290	0.098
Phosphatidylcholine (16:0_18:2) levels		0.026	0.253	8.276	0.219
Phosphatidylcholine (16:0_18:3) levels		−0.165	0.870	4.396	0.111
Phosphatidylcholine (16:0_20:2) levels		0.025	0.341	2.585	0.630
Phosphatidylcholine (16:1_18:2) levels		0.016	0.362	2.662	0.850
Phosphatidylcholine (17:0_18:2) levels		0.035	0.566	0.719	0.698
Phosphatidylcholine (18:0_18:2) levels		0.013	0.830	7.725	0.102
Phosphatidylcholine (18:0_20:4) levels		−0.009	0.703	2.936	0.402
Phosphatidylcholine (18:1_18:1) levels		0.031	0.571	4.183	0.242
Phosphatidylcholine (18:1_18:2) levels		−0.007	0.717	3.478	0.481
Phosphatidylcholine (18:1_20:2) levels		0.009	0.834	1.592	0.451
Phosphatidylcholine (18:2_18:2) levels		0.035	0.466	2.353	0.308
Phosphatidylethanolamine (16:0_18:2) levels		0.020	0.599	5.468	0.361
Phosphatidylinositol (18:0_18:1) levels		−0.103	0.317	5.696	0.223
Phosphatidylinositol (18:0_18:2) levels		0.028	0.555	2.435	0.487
Phosphatidylinositol (18:0_20:3) levels		−0.013	0.635	1.363	0.714
Sterol ester (27:1/18:3) levels	SVS	0.016	0.669	0.552	0.759
Sterol ester (27:1/20:3) levels		0.011	0.812	0.341	0.843
Phosphatidylcholine (18:0_20:3) levels		−0.014	0.770	0.235	0.889
Phosphatidylcholine (O-16:1_20:3) levels		0.004	0.945	0.099	0.952
Sphingomyelin (d32:1) levels	CS	0.001	0.954	0.993	0.803
Sphingomyelin (d40:2) levels		0.005	0.843	0.167	0.920
Phosphatidylcholine (18:2_0:0) levels	FSC-A on granulocyte	−0.116	0.663	0.404	0.817
Sphingomyelin (d40:2) levels	CD8 on CD39^+^ CD8^+^ T cell	0.064	0.215	7.588	0.180
CD14-CD16-absolute count	LAS	−0.094	0.449	1.675	0.433
CD3 on naive CD8^+^ T cell		−0.006	0.816	0.273	0.965
CD3 on CD45RA^+^ CD4^+^ T cell		−0.005	0.855	0.414	0.937
CD40 on CD14^+^ CD16-monocyte		−0.007	0.872	1.215	0.545
CD39 on granulocyte		−0.014	0.948	1.912	0.384
SSC-A on granulocyte		−0.018	0.659	2.121	0.548
CD62L-monocyte %monocyte	SVS	−0.036	0.837	2.192	0.334
BAFF-R on IgD^+^ CD38-naive B cell		0.008	0.696	0.456	0.928
BAFF-R on IgD^+^ CD38-unswitched memory B cell		−0.010	0.813	1.424	0.491
BAFF-R on IgD^+^ CD38^+^ B cell		0.001	0.927	0.574	0.966
BAFF-R on IgD^+^ CD38dim B cell		0.008	0.675	0.405	0.939
BAFF-R on IgD-CD24-B cell		−0.011	0.530	2.315	0.510
BAFF-R on IgD-CD27-B cell		−0.011	0.533	2.258	0.521
BAFF-R on transitional B cell		0.009	0.674	0.417	0.937
BAFF-R on B cell		0.007	0.666	0.404	0.982
CD33 on granulocytic myeloid-derived suppressor cells		0.005	0.831	0.684	0.710
HLA DR on B cell		0.010	0.724	0.494	0.781
CD28^+^ CD45RA^+^ CD8^+^ T cell absolute count	CS	−0.086	0.416	1.954	0.376
CD45RA^+^ CD28-CD8^+^ T cell absolute count		0.005	0.636	39.417	0.541
BAFF-R on IgD^+^ CD24-B cell		−0.002	0.903	1.976	0.853
BAFF-R on IgD^+^ CD38-B cell		−0.010	0.624	1.834	0.766
BAFF-R on IgD^+^ CD38-naive B cell		−0.004	0.851	1.968	0.742
BAFF-R on IgD^+^ CD38^+^ B cell		0.012	0.422	4.228	0.517
BAFF-R on IgD^+^ CD38dim B cell		−0.002	0.911	2.064	0.840
BAFF-R on naive-mature B cell		−0.003	0.856	2.062	0.840
BAFF-R on transitional B cell		−0.007	0.741	1.727	0.786
CD25 on naive-mature B cell		−0.001	0.974	0.043	0.998

### 3.2 Causal associations between immune cell phenotypes and IS subtypes

Recognizing the significant involvement of immune cells in the pathogenesis of IS subtypes, our study sought to investigate the causal association between 731 immune cell phenotypes and IS subtypes. Employing criteria akin to those used for lipid species, we meticulously identified instrumental variables for these immune cell phenotypes. Out of 731 phenotypes, we successfully identified 614 species with eligible IVs, each characterized by *F*-statistic values surpassing 10, ranging from 29.85 to 5062.70 (Supplementary Table S6).

**Figure 3:**
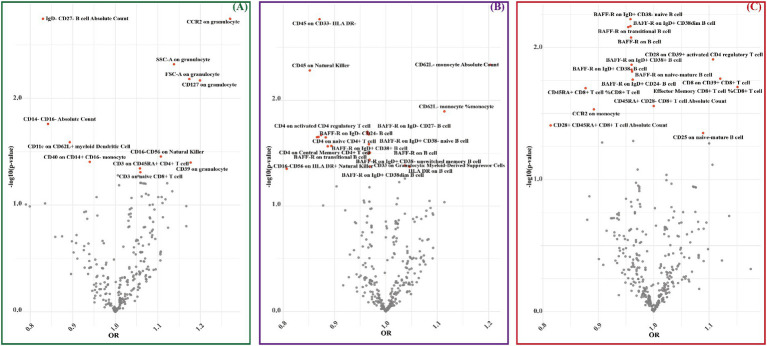
MR estimates for immune cell phenotypes and IS subtypes (LAS, SVS, and CS) were assessed by IVW. **(A)** The significant causal effect of immune cell phenotypes on LAS. **(B)** The significant causal effect of immune cell phenotypes on SVS. **(C)** The significant causal effect of immune cell phenotypes on CS. The dots colored in red indicate significant estimates by IVW (*p* < 0.05).

MR results unveiled genetic causal associations between 12 immune cell phenotypes and LAS, with 8 showing positive correlations and 4 showing negative correlations (Figure 3A and Supplementary Table S7). Similarly, 21 immune cell phenotypes were linked to SVS, with 4 exhibiting positive correlations and 17 displaying negative correlations (Figure 3B and Supplementary Table S16). Furthermore, 16 immune cell phenotypes demonstrated genetic causal associations with CS, with 4 manifesting positive correlations and 12 exhibiting negative correlations (Figure 3C and Supplementary Table S25).

Consistently, the *p*-values of MR-Egger intercept estimates exceeded 0.05, indicating the absence of significant pleiotropy bias. Additionally, the Cochran *Q* test yielded *p*-values greater than 0.05 across all analyses, reinforcing the lack of heterogeneity and thereby affirming the robustness of our causal inferences (see Table 1; Supplementary Tables S8, S9, S17, S18, S26, S27).

### 3.3 Immune cell-mediated pathways linking lipidomic profiles to IS subtypes

To explore the potential role of immune cell phenotypes as mediators in the causal pathway between lipidomic profiles and ischemic stroke subtypes, we employed TSMR approach. Specifically, we focused on lipids associated with LAS, SVS, and CS, and assessed their MR-estimated effects against immune cell phenotypes robustly associated with each subtype.

**Figure 4:**
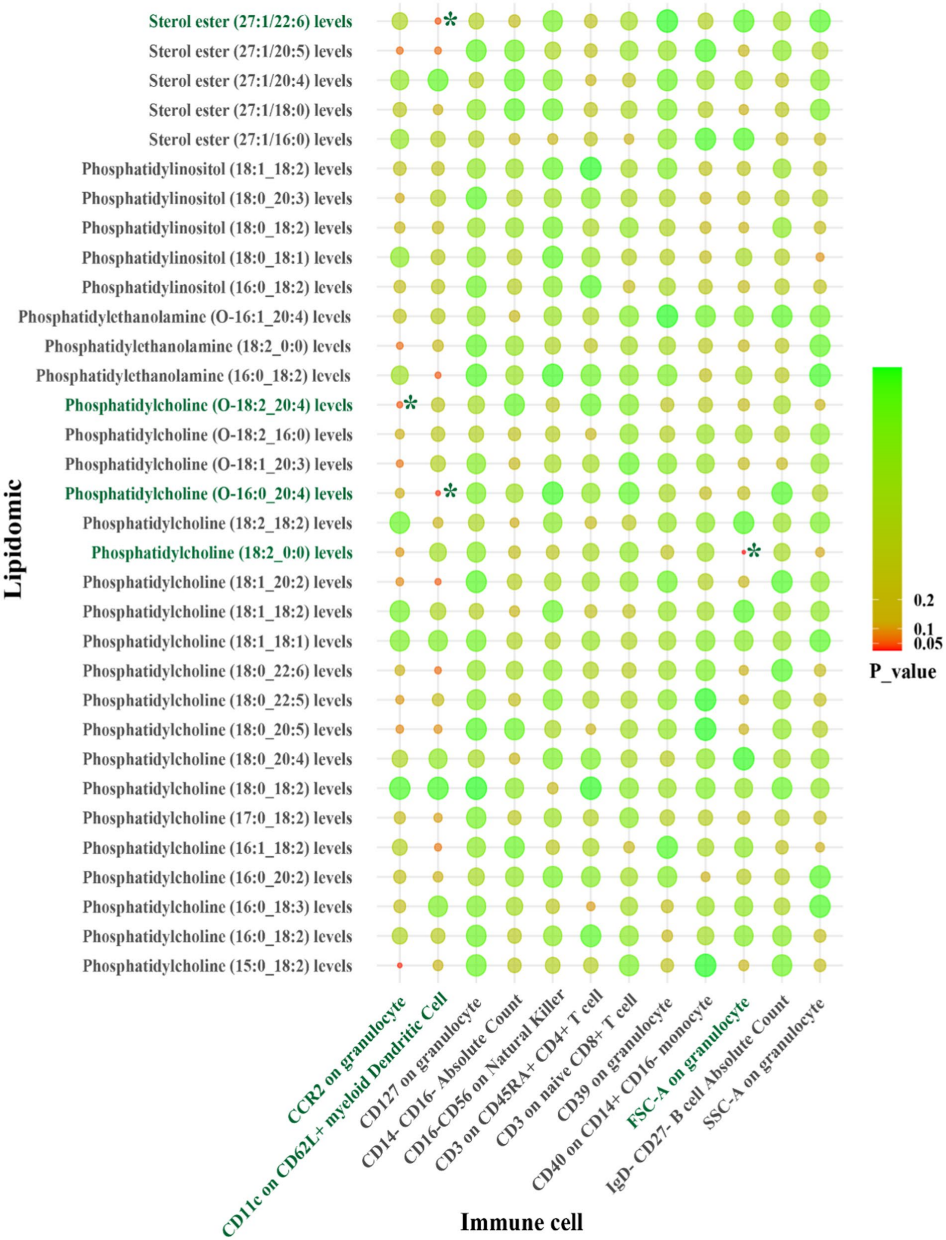
IVW results from MR analyses of lipids significantly causally associated with LAS, and immune cell phenotypes significantly causally associated with LAS. *p*-values for both IVW and WM analyses <0.05, marked as *.

**Figure 5:**
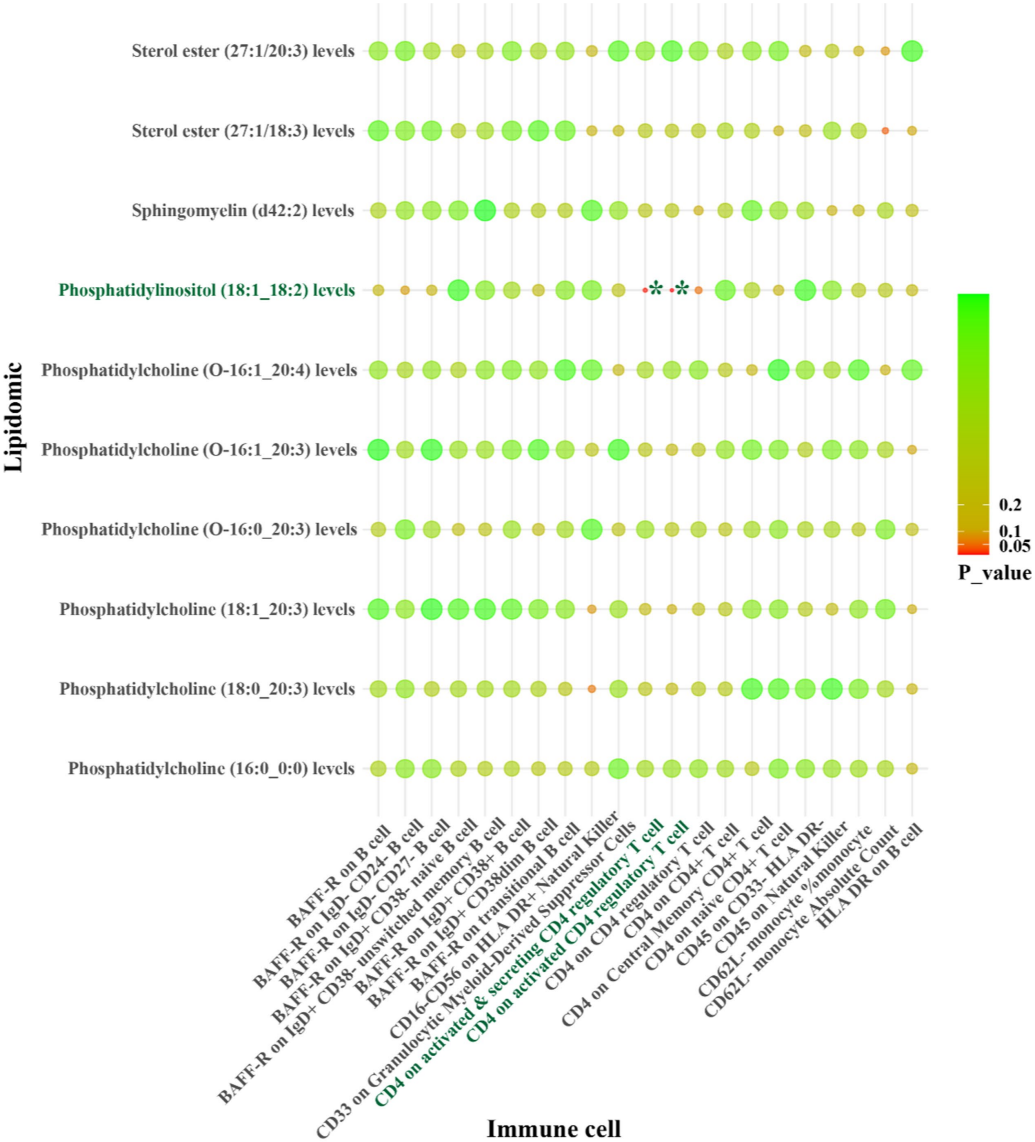
IVW results from MR analyses of lipids significantly causally associated with SVS, and immune cell phenotypes significantly causally associated with SVS. *p*-values for both IVW and WM analyses <0.05, marked as *.

**Figure 6:**
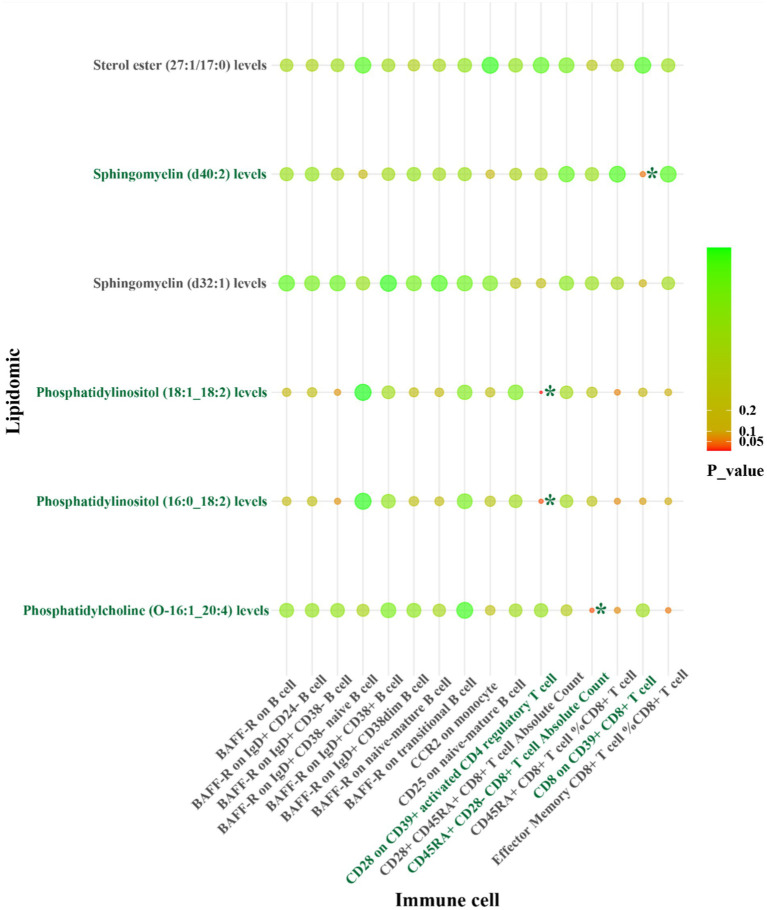
IVW results from MR analyses of lipids significantly causally associated with CS, and immune cell phenotypes significantly causally associated with CS. *p*-values for both IVW and WM analyses <0.05, marked as *.

For LAS, our analysis revealed inverse genetic correlations between three lipid phenotypes (sterol ester (27:1/22:6) levels, phosphatidylcholine (O-16:0_20:4) levels, and phosphatidylcholine (18:2_0:0) levels) and CD11c on CD62L^+^ myeloid dendritic cells, as well as FSC-A on granulocytes (*β*_IVW_: −0.406 to −0.121). Additionally, phosphatidylcholine (O-18:2_20:4) levels were positively correlated with CCR2 on granulocytes (*β*_IVW_: 0.300) (Figure 4 and Supplementary Table S10). Regarding SVS, phosphatidylinositol (18:1_18:2) levels exhibited positive correlations with CD4 on activated CD4 regulatory T cells and CD4 on activated & secreting CD4 regulatory T cells, with *β*_IVW_ values of 0.408 and 0.383, respectively (Figure 5 and Supplementary Table S19). In the case of CS, sphingomyelin (d40:2) levels were inversely correlated with CD8 on CD39^+^ CD8^+^ T cells (*β*_IVW_: −0.209). Furthermore, phosphatidylcholine (O-16:1_20:4) levels, phosphatidylinositol (16:0_18:2) levels, and phosphatidylinositol (18:1_18:2) levels showed positive correlations with CD45RA^+^ CD28-CD8^+^ T cell absolute count and CD28 on CD39^+^ activated CD4 regulatory T cells (*β*_IVW_: 0.343 to 37.991) (Figure 6 and Supplementary Table S28). No pleiotropy bias and heterogeneity were found by MR-Egger intercept estimates and Cochran *Q* test (Table 1; Supplementary Tables S11, S12, S20, S21, S29, S30).

A summary of STMR estimates revealed six robust causal pathways linking lipid levels, immune cell phenotypes, and IS subtypes. These pathways exhibited consistent directions of total, direct, and indirect effects. Three pathways involving phosphatidylcholine (O-18:2_20:4), phosphatidylcholine (O-16:0_20:4), and sterol ester (27:1/22:6) levels were positively associated with LAS, mediated by CCR2 on granulocytes and CD11c on CD62L^+^ myeloid dendritic cells. Specifically, higher levels of phosphatidylcholine (O-18:2_20:4) and CCR2 on granulocytes correlated with increased LAS risk. Similarly, elevated levels of phosphatidylcholine (O-16:0_20:4) and sterol ester (27:1/22:6), along with lower levels of CD11c on CD62L^+^ myeloid dendritic cells, were associated with increased LAS risk.

Conversely, other pathways involving phosphatidylcholine (18:2_0:0) and phosphatidylinositol (18:1_18:2) levels were inversely related to LAS and SVS, mediated by FSC-A on granulocytes, CD4 on activated CD4 regulatory T cells and CD4 on activated & secreting CD4 regulatory T cells. Higher levels of phosphatidylcholine (18:2_0:0) and lower levels of FSC-A on granulocytes correlated with a decreased risk of LAS. Similarly, higher levels of phosphatidylinositol (18:1_18:2) with lower levels of CD4 on activated CD4 regulatory T cells and CD4 on activated & secreting CD4 regulatory T cells were associated with a decreased risk of SVS. Detailed *β* values of the MR estimates are provided in Table 2.

**Table 2:** Two-step Mendelian randomization analyses of the causal effects between lipidomic, immune cell phenotypes, and ischemic stroke of LAS, SVS, and CS.

Exposure	Mediator	Outcome	Total effect (*β*_0_)	Direct effect (*β*_0_ − *β*_1_*β_2_)	Indirect effect (*β*_1_*β_2)_	Proportion mediated (%)
Phosphatidylcholine (18:2_0:0) levels	FSC-A on granulocyte	LAS	−0.381	−0.316	−0.065	17.05
Phosphatidylcholine (O-18:2_20:4) levels	CCR2 on granulocyte	LAS	0.265	0.193	0.072	27.06
Phosphatidylcholine (O-16:0_20:4) levels	CD11c on CD62L^+^ myeloid Dendritic Cell	LAS	0.181	0.163	0.018	10.05
Sterol ester (27:1/22:6) levels	CD11c on CD62L^+^ myeloid Dendritic Cell	LAS	0.312	0.283	0.029	9.34
Phosphatidylinositol (18:1_18:2) levels	CD4 on activated CD4 regulatory T cell	SVS	−0.156	−0.101	−0.055	35.05
Phosphatidylinositol (18:1_18:2) levels	CD4 on activated & secreting CD4 regulatory T cell	SVS	−0.156	−0.101	−0.055	35.35
Phosphatidylcholine (O-16:1_20:4) levels	CD45RA^+^ CD28-CD8^+^ T cell absolute count	CS	−0.193	−0.195	0.002	NA
Phosphatidylinositol (16:0_18:2) levels	CD28 on CD39^+^ activated CD4 regulatory T cell	CS	−0.22	−0.255	0.035	NA
Phosphatidylinositol (18:1_18:2) levels	CD28 on CD39^+^ activated CD4 regulatory T cell	CS	−0.161	−0.201	0.040	NA
Sphingomyelin (d40:2) levels	CD8 on CD39^+^ CD8^+^ T cell	CS	0.132	0.156	−0.024	NA

*β*, beta; *β*_0_ is the causal effect of exposure on the outcome; *β*_1_ is the causal effect of the mediator on the outcome; *β*_2_ is the causal effect of mediator on outcome; indirect effect (*β*_1_*β_2_) is the effect of exposure on outcome via corresponding mediator; proportion mediated is calculated as the “indirect effect/total effect”; LAS, large artery stroke; SVS, small vessel stroke; SC, cardioembolic stroke; NA, data not available.

**Figure 7:**
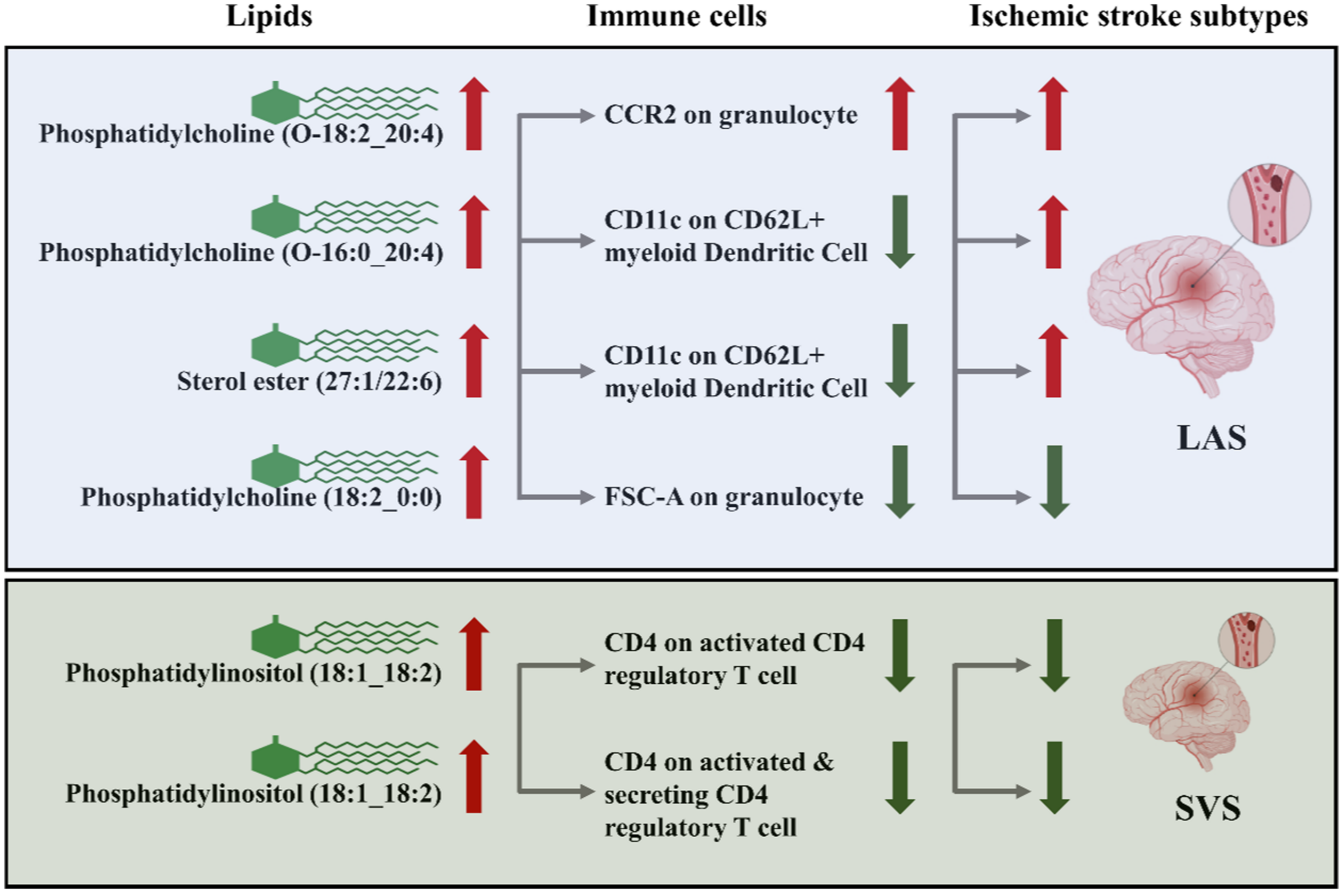
CCR2 on granulocytes, CD11c on CD62L^+^ myeloid dendritic cells, FSC-A on granulocytes, CD4 on activated CD4 regulatory T cells, and CD4 on activated & secreting CD4 regulatory T cells as immune cell mediators in the lipid-LAS/SVS causal pathways. The arrows represent the direction of lipids or immune cell levels and the risk effect of IS subtypes. For example, when phosphatidylcholine (O-18:2_20:4) and CCR2 on granulocyte levels are elevated, LAS risk is increased.

In summary, CCR2 on granulocytes, CD11c on CD62L^+^ myeloid dendritic cells, and FSC-A on granulocytes are identified as potential mediators in the lipid-LAS causal pathways. Levels of CD4 on activated CD4 regulatory T cells and CD4 on activated & secreting CD4 regulatory T cells are identified as potential mediators in the lipid-SVS causal pathways (Figure 7).

## 4 Discussion

We conducted STMR analysis to explore the causal relationship between lipidomic profiles, immune cell phenotypes, and the risk of three distinct types of IS. Our investigation revealed that phosphatidylcholine (O-18:2_20:4), phosphatidylcholine (O-16:0_20:4), and sterol ester (27:1/22:6) levels are associated with an increased risk of LAS. These associations are mediated through the CCR2 on granulocytes and CD11c on CD62L^+^ myeloid dendritic cells. Moreover, we identified phosphatidylcholine (18:2_0:0) levels as a protective factor against LAS, mediated by FSC-A on granulocytes. Additionally, phosphatidylinositol (18:1_18:2) levels emerged as a protective factor against SVS, mediated by CD4 on activated CD4 regulatory T cells and CD4 on activated & secreting CD4 regulatory T cells. Importantly, our analyses revealed no significant heterogeneity or evidence of horizontal pleiotropy in the data.

First, our findings establish a robust causal relationship between specific lipid species and LAS, SVS, and cardioembolic stroke (CS), with 35 out of 179 lipid species being genetically associated with LAS. Phosphatidylcholines and sterol esters were significant contributors to LAS risk. Similarly, 10 out of 179 lipid species were genetically associated with SVS, primarily phosphatidylinositol (18:1_18:2). This observation expands our understanding of lipid metabolism’s role in IS beyond traditional markers like total cholesterol, LDL-C, and HDL-C, highlighting specific lipid molecules’ crucial roles in IS development.

Phosphatidylcholines, phosphatidylinositol, and sterol esters containing different fatty acids have been less emphasized in IS risk assessment models. However, their significant association with LAS, SVS, and CS in our study points towards their potential role in IS pathophysiology. These lipids are critical components of cell membranes and lipoproteins, and alterations in their composition have been linked to changes in lipoprotein functionality and signaling (23–25). Phosphatidylcholine, phosphatidylinositol, and sterol esters are crucial components of membrane lipids, with many essential cellular processes depending heavily on their interactions. The membrane hypothesis suggests that dysfunction in membrane lipids may contribute to the development of diseases such as schizophrenia, Alzheimer’s disease, autoimmune disorders, chronic fatigue syndrome, and cancer. The concept that cell membranes contain transient microdomains with distinct lipid compositions has led to the development of selective lipid-targeted therapies, known as membrane lipid therapy (26, 27). Lipid analogs such as perifosine, plasmalogens, and edelfosine have been developed for the treatment of solid tumors, hematological malignancies, and neurodegenerative diseases (28). Recent evidence suggests that unconventional lipids, including phosphatidylcholine, phosphatidylethanolamine, phosphatidylinositol, phosphatidylserine, and sphingomyelin, are crucial in IS development (29–32). Our genetic informatics-driven identification of specific lipid profiles associated with IS risk may provide more accurate predictions due to their effects on inflammation, endothelial function, and plaque stability.

For the first time, sterol ester and phosphatidylinositol levels were established as significant causal risk factors for LAS and SVS, respectively. The risk of LAS increased by approximately 37% for each unit change in sterol ester (27:1/22:6), while the risk of SVS decreased by approximately 15% for each unit change in phosphatidylinositol (18:1_18:2). These unbiased results strengthen the genetic evidence beyond observational studies, emphasizing the intricate role of lipids in cerebrovascular disease beyond traditional pathways. While elevated LDL-C and HDL-C levels are well-established risk factors for IS, our study suggests that sterol ester (27:1/22:6) and phosphatidylinositol (18:1_18:2)'s specific role in LAS and SVS pathogenesis may involve complex interactions with immune cell pathways. Subclinical inflammation contributes to endothelial dysfunction and the buildup of immune-active cells in the vessel walls. These immune cells and lipids are crucial in forming and growing atherosclerotic lesions, leading to IS (11, 33, 34).

Our investigation into immune cell phenotypes revealed that CCR2 on granulocytes, CD11c on CD62L^+^ myeloid dendritic cells, and FSC-A on granulocytes are genetically associated with LAS. Additionally, CD4 on activated CD4 regulatory T cells is genetically associated with SVS. Granulocytes, particularly neutrophils, are crucial in the pathophysiology of ischemic stroke, where they release neurotoxic agents such as reactive oxygen species (ROS) and matrix metalloproteinases (MMPs). These agents contribute to the disruption of the blood–brain barrier, thereby exacerbating tissue damage. Furthermore, neutrophils form neutrophil extracellular traps (NETs), which promote thrombosis and impede thrombolysis, complicating the ischemic injury (35). Dendritic cells are instrumental in antigen presentation within the ischemic brain. By presenting central nervous system (CNS) antigens to T cells, they amplify immune responses and drive inflammation. The interaction between dendritic cells and T cells, mediated by molecules such as MHC class II, is critical in the progression of post-stroke inflammation (36). These findings enrich the current understanding of immune cells’ role in IS by identifying specific pathways that may contribute to IS pathogenesis.

Phosphatidylcholine mitigates the adverse effects of immune cell-mediated neuroinflammation on neuronal differentiation and plasticity. By modulating the inflammatory response, phosphatidylcholine enhances neuronal survival and proper differentiation, positioning itself as a potential therapeutic agent in cases of neuronal dysfunction arising from lipid-immune interactions. Additionally, bioactive lipids such as lysophosphatidylcholine (LPC) play a pivotal role in mediating the interaction between immune cells and apoptotic cells during efferocytosis. LPC acts as a “Find-Me” signal, guiding phagocytes to the site of inflammation, thereby facilitating the efficient clearance of apoptotic cells, which is crucial for resolving inflammation and promoting tissue repair. These findings reinforce the critical relationship between lipids, immune cells, and ischemic stroke, highlighting the potential therapeutic implications of targeting lipid-immune interactions in stroke treatment (9, 37–39). Based on the results of this study, it can be speculated that the prognosis of LAS may be improved by reducing the plasma levels of Phosphatidylcholine (O-18:2_20:4), Phosphatidylcholine (O-16:0_20:4), and Sterol ester (27:1/22:6). Conversely, increasing the levels of Phosphatidylcholine (18:2_0:0) may enhance the prognosis of LAS. Additionally, elevating the levels of Phosphatidylinositol (18:1_18:2) could potentially improve the prognosis of SVS. Additionally, the prognosis of ischemic stroke may be improved by modulating the activity of immune cells, such as granulocytes and myeloid dendritic cells.

The mediation analysis provided intriguing insights into how lipid levels could influence LAS and SVS risk through immune cell processes. We identified potential pathways whereby specific lipids might modulate LAS and SVS risk by altering immune cell counts, such as granulocytes, myeloid dendritic cells, and T cells. The absence of evidence for directional horizontal pleiotropy in our results supports the potential causal relationship between identified lipids and immune cell phenotypes with LAS and SVS. By using genetic instrumental variables (IVs) to infer causality, we circumvent the limitations of observational studies that can be confounded by lifestyle factors and reverse causation. This methodological strength enhances the reliability of our findings and provides a stronger basis for developing intervention strategies based on genetic susceptibilities (19, 40).

While our study marks a significant step forward, further research is needed to elucidate the biological mechanisms through which these identified lipids and immune cells influence LAS and SVS risk. Experimental studies in cellular and animal models could provide deeper insights into the pathophysiological processes involved. Our findings identified immune cell phenotypes that may mediate the relationship between lipid levels and ischemic stroke subtypes. However, the mediation analysis was constrained by the available data, limiting our ability to capture all potential mediators or interactions. Future research should aim to include a broader spectrum of immune cell phenotypes. Additionally, clinical trials designed to modulate these specific lipid and immune cell pathways could validate the therapeutic potential of our findings. Including populations from diverse ethnic backgrounds could enhance the generalizability of our findings and uncover population-specific genetic risk factors for IS.

In conclusion, our study reveals an intricate landscape of genetic factors influencing LAS and SVS risk, involving specific lipid species and immune cells. These findings not only advance our understanding of IS pathogenesis but also point toward novel therapeutic targets that could transform IS management. As we move towards a more personalized medicine approach, integrating genetic risk factors with clinical strategies will be crucial in combating the global burden of ischemic stroke.

## 5 Conclusion

This study identifies significant genetic associations between specific lipids—namely phosphatidylcholine, sterol ester, and phosphatidylinositol—and the risk of LAS and SVS. Additionally, we identified key immune cell phenotypes that contribute to LAS and SVS risk and act as mediators in the lipid-LAS/SVS causal pathway. These findings enhance our understanding of the genetic factors influencing LAS and SVS pathophysiology and suggest potential targets for therapeutic intervention. This work advances the current knowledge by providing new insights into the complex interactions between lipid metabolism, immune response, and ischemic stroke risk, highlighting novel avenues for treatment strategies.

## Data Availability

The original contributions presented in the study are included in the article/Supplementary material, further inquiries can be directed to the corresponding authors.

## Footnotes

^1^ https://www.ebi.ac.uk/gwas/
